# Ghost-rocks’ microbiota: metagenomic insights into their influence on the biogeochemistry of karstic cave and groundwater

**DOI:** 10.1093/femsec/fiag047

**Published:** 2026-06-10

**Authors:** Guillaume Peugnet, Céline Pisapia, Bénédicte Ménez, Maud Watkinson, Léna Lecourt, Nicolas Peugnet, Julien Bouchez, Laurent Bruxelles, Emmanuelle Gérard

**Affiliations:** Université Paris Cité, Institut de physique du globe de Paris, CNRS, 75005 Paris, France; Université Paris Cité, Institut de physique du globe de Paris, CNRS, 75005 Paris, France; Université Paris Cité, Institut de physique du globe de Paris, CNRS, 75005 Paris, France; Université Paris Cité, Institut de physique du globe de Paris, CNRS, 75005 Paris, France; BRGM, F-45060 Orléans, France; Université Paris Cité, Institut de physique du globe de Paris, CNRS, 75005 Paris, France; Sorbonne Université, CNRS, LIP6, F-75005 Paris, France; Université Paris Cité, Institut de physique du globe de Paris, CNRS, 75005 Paris, France; Université Jean Jaurès, TRACES, 31058 Toulouse, France; Université Witwatersrand, Johannesburg, South Africa; Université Paris Cité, Institut de physique du globe de Paris, CNRS, 75005 Paris, France

**Keywords:** cave microbiology, metagenomics, karst, porin-cytochrome complexes, atmospheric chemosynthesis

## Abstract

Microbial communities in the critical zone drive key geochemical processes, but many subsurface habitats remain poorly characterized. Ghost-rock karst systems in particular represent unexplored microbial niches. Here, we provide the first genome-resolved metagenomic comparison of ghost-rock and groundwater microbial communities from the Sterkfontein karst system (South Africa). Ghost-rock and groundwater communities host distinct taxonomic and metabolic assemblages. Groundwater communities are dominated by chemolithotrophs capable of oxidizing sulfur- and nitrogen-bearing compounds, and by heterotrophs degrading refractory, plant-derived organic matter. In contrast, primary producers in ghost-rocks likely rely on atmospheric chemosynthesis via trace gas oxidation, while glycogen metabolism and necromass recycling point to adaptations to oligotrophic and fluctuating hydrological conditions. Groundwater taxa with metal-interacting pathways may initiate bedrock colonization via metal oxidation, whereas ghost-rock communities include potential metal reducers that could drive iron and manganese oxide dissolution and influence trace element mobility. Together, these results underscore ghost-rocks as active microbial and geochemical hot spots within karst systems that may play a non-negligible role on biomineralization/bioweathering processes and on shaping (sub)terrestrial landscapes and global biogeochemical cycles.

## Teaser

Metagenomics of the Sterkfontein ghost-rock karst microbiota reveals their likely role in bioweathering and biogeochemical cycles.

## Introduction

Subsurface ecosystems host most of Earth’s micro-organisms (Bar-On et al. 2018), whose activity can significantly influence critical zone (CZ) processes, including rock weathering (Akob and Küsel 2011, Wild et al. 2022). Subsurface ecosystems, such as fractured aquifers, are spatially heterogeneous and dynamic over time, generated as water circulates through fracture networks (Bochet et al. 2020). Significant differences exist between planktonic communities in groundwater and the biofilm communities attached to rocks (Flynn et al. 2013, Lazar et al. 2019), and these communities change in response to environmental cues (Yan et al. 2021). It is thus a major challenge to integrate these spatial and temporal heterogeneities when one seeks to understand processes that influence the dynamics of subsurface environments, particularly in the deeper part of the CZ. Consequently, the contribution of planktonic and biofilm communities to major biogeochemical processes in the deep CZ remain poorly understood (Smith et al. 2018).

In the shallow subsurface, carbonate karst systems are an important component of the CZ covering around 15% of the Earth’s ice-free continents and forming complex cave networks (Covington et al. 2023). Caves can be classified according to their lithology and speleogenetic process (e.g. lava tubes caves, epigenic karstic carbonate caves, or hypogenic carbonate caves formed by sulfuric acid speleogenesis). From a microbiological perspective, non-karstic caves such as lava tubes may be comparable to epigenic karstic caves regarding organic carbon availability, and energy sources for microbial community.

Estimates suggest that only 0.02%–1.3% of micro-organisms in carbonate karstic cave are cultivable, depending on the methodology used (Bender et al. 2020). Microbial ecology studies of oligotrophic carbonate caves are limited in part due to very low biomass, with between 10^3^ and 10^5^ cells/ml in karst springs and cave lakes (Hershey and Barton 2018), and around 10^6^ cells per gram of rocks (Barton et al. 2006). After the pioneer works in cave microbiology that offered the first insights in the cave microbiota composition and structure (Barton et al. 2004), metabarcoding and metagenomics have revealed a distinct cave microbiota, compared to surface soils (Ortiz et al. 2013). Sediments and biofilms on cave walls are dominated by Pseudomonadota, Actinobacteriota, and Acidobacteriota, whereas groundwater communities are dominated by Pseudomonadota, Verrucomicrobiota, and Patescibacteriota (Turrini et al. 2024). Shotgun metagenomics of speleothems (Ortiz et al. 2014), sediment (Wiseschart et al. 2019), or cave wall biofilms (Turrini et al. 2020) have revealed metabolic diversity and the importance of autotrophic organisms in these ecosystems. Microbial activity has been shown to influence greenhouse gas cycling (Martin-Pozas et al. 2022), and nutrient and elemental cycling, particularly of iron and manganese (Carmichael and Bräuer 2015).

A microbial origin has been suggested for ferromanganese minerals often encountered as crusts on cave’ walls (Spilde et al. 2005, Gázquez et al. 2011, Fonollá et al. 2020), stromatolite-like Mn patinas (Bernardini et al. 2021), and sediments or speleothems (Kotula et al. 2019). These oxides are thought to form via chemolithoautotrophic organisms that would mine reduced elements present in the bedrock and oxidize them to gain energy, therefore contributing to rock weathering. While iron-based chemolithoautotrophy has long been described (Winogradsky 1888), manganese-based chemolithoautotrophy is more recently documented (Yu and Leadbetter 2020). Alternative heterotrophic pathways for manganese oxides formation, involving reactive oxygen species (ROS) production, or multicopper oxidases (MCOs) have also been proposed (Carmichael and Bräuer 2015).

In this context, caves formed via ghost-rock karstification are of high interest for studying the interplay between microbial activity and mineral weathering. This new model of karstification is a two step process with (i) an isovolumetric chemical weathering of the bedrock that takes place under low hydrodynamic conditions: the bedrock, when submerged, undergoes dissolution of soluble ions, while leaving behind a residual alterite (the insoluble fraction), highly porous (up to 65% porosity), called ghost-rock; (ii) physical erosion triggered either by the lowering of the saturated zone, inducing an energetic water-flow capable of removing ghost-rock, or by the gravitational collapse of ghost-rock masses, thus creating caves and underground rivers characteristic of karstic systems (Dubois et al. 2014). Interestingly and following this multi-stage karstification process, some pockets of ghost-rocks may nevertheless remain in place without being completely emptied, representing considerable volumes of weathered rock, far greater than previously thought (e.g. up to 200 million m^3^ in the French Larzac system, Baral et al. 2024). They would thus constitute a largely unexplored component of karst systems, and their implications for the functioning of these karsts remain to be elucidated. In particular, due the high porosity of the residual alterite, they may act as hidden water reservoirs in the vadose zone of karsts (Champollion et al. 2018, Durand et al. 2025), contributing to global water cycle and more specifically to the drinking water resources of populations. However, the high porosity and water content of these ghost-rocks pockets make them ideal habitats for micro-organisms. Then, they might also be considered as putative niches for subsurface microbial ecosystems in which they may influence mineral weathering and/or biomineralization processes, either in bedrocks or in alterites, playing then a role on elemental (re)mobilization along the karst history. In particular, the identification of secondary metallic oxides (Fe, Mn) in these alterites, of which a microbial origin has already been proposed (Dandurand et al. 2014, Dubois et al. 2014, Pisapia et al. 2021) raises the question of the impact of this putative microbial activity on rock weathering and metal cycling at depth, and on its broader influence on global metal biogeochemical cycles. However, to date, the microbial metabolic potential in ghost-rocks has never been studied.

The main objective of this study was then to investigate what kind and to which extent microbial ecosystems of a ghost-rock karst system may impact rock weathering/mineralization processes and influence the geochemistry of groundwaters, notably through metal cycling. For this purpose, we focused on the Sterkfontein karst system (South Africa) that presents one of the most impressive volume of ghost-rocks ever described (Bruxelles 2018). These alterites were preliminarily described to host large amounts of iron and manganese oxides jointly with the presence of micro-organisms whose metabolisms were still unknown (Pisapia et al. 2021). The objectives were then to achieve the first exhaustive description of the microbial ecology of this ghost-rock system, including alterites and groundwaters, and to highlight the main metabolic potentials at stake in these compartments of the cave system. Based on the working hypothesis that microbially-induced metal cycling might be central in these environments, either *via* the oxidation of trace metals from the dolomitic bedrocks as proposed by Spilde et al. (2005) or *via* the reduction of metal-oxides from the alterites as electron acceptors, we then specifically focused on the description of microbial metabolic potentials linked with iron and manganese cycling. To answer these questions, we used genome-resolved shotgun metagenomics, alongside with geochemical analyses of the studied caves. Notably, we used a global approach at the level of reads, contigs and MAGs, with a particular focus on metal-interacting pathways, including metallophores biosynthetic gene clusters (BGCs), multicopper oxidases (MCOs), and extracellular electron transfer (EET) systems.

## Methods

### Study site

**Figure 1:**
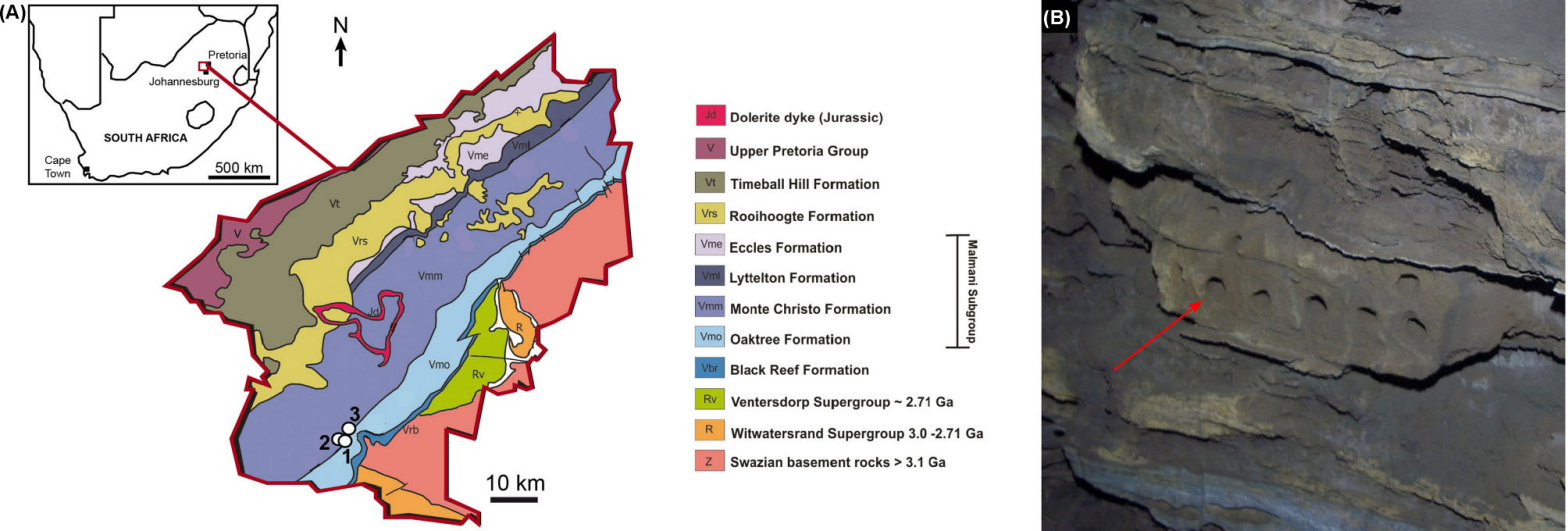
Sampling site localization and description.(A) Simplified geological map of the Cradle of Humankind, NW of Johannesburg, developed in the Transvaal Supergroup (modified from Makhubela et al. 2017). The location of the three studied caves, Sterkfontein Cave (1), Fault Cave (2), and Glover’s Cave (3) is provided. (B) Photograph of a typical outcrop of porous ghost-rock in Fault Cave, sampled using 50 ml Falcon^TM^ tubes (visible as holes, red arrow). The outcrop exhibits ghost-rock developed through alteration of dolomitic beds, with residual chert layers forming more resistant horizons. Thin, millimetric, siliceous partitions are visible within ghost-rock pockets, contributing to the structuring and stability of the outcrop.

The ghost-rock karst system of Sterkfontein in South Africa is located 35 km northwest of Johannesburg, and is part of the Cradle of Humankind UNESCO World heritage site (Fig. 1A). It develops in metric to pluri-metric dolomitic beds from the Malmani Subgroup (2.64–2.50 Gy), intercalated with centimetric chert beds. The system is highly labyrinthine, extending for more than 5 km, mostly along bedrock fractures, and harbors more than 400 caves (Bruxelles 2018). A tectonic uplift during the Oligocene led to incision by the Blaubank river, which, despite a low incision rate (down to 3 m per My throughout the Neogene and Quaternary), progressively removed ghost-rock, and opened the present cave system (Bruxelles 2018). This karst system presents one of the most important ghost-rock example ever discovered, with large volumes of ghost-rock pockets, in place in the caves (Fig. 1B) including at the contact with the water table, and easily accessible. It was then an ideal system where performing a first metagenomic study of these peculiar environments.

In that perspective, three caves from this wide karstic system were investigated: Sterkfontein Cave, Fault Cave and Glover’s Cave (Fig. 1A). Sterkfontein and Fault Caves belong to the same network, the first one being currently open to tourism, while Fault cave, not accessible to the public, can be considered as a more pristine environment. Glover’s Cave, located a few kilometers away in the Kromdraai system, but within the same geological context, was also investigated for representativity (Fig. 1A).

### Sampling

All samples analyzed in this study (Supplementary Table S1) were collected during a field campaign in 2019. To investigate the microbial ecology of the karst system, both groundwater and ghost-rocks were sampled for metagenomic analyses in the targeted caves.

For each cave, 15 l of groundwater were collected at the water table using 5 l plastic bottles rinsed three times beforehand with *in situ* cave groundwater in order to limit external contamination. Groundwater samples were then filtered on the day of collection, using vacuum-pump filtration systems. All samples were first filtered through 3 µm Whatman^®^ filters to remove large particles and aggregates followed by 0.2 µm Whatman^®^ filters to recover microbial cells. Both sets of filters were then stored at −20°C until return to the laboratory and subsequent DNA extraction. During filtration, 500 ml of filtrate from each cave were collected in 100 ml Nalgene^TM^ bottles (that were previously cleaned with three successive nitric acid wash) and acidified using a drop of 7 N nitric acid (HNO_3_) for triplicated elemental analyses.

Ghost-rock samples were collected from each cave walls using 50 ml Falcon^TM^ tubes, easily inserted due to the loose nature of the ghost-rock (Fig. 1B). The tubes were directly stored at −20°C until return to the laboratory for subsequent DNA extraction, and selected ghost-rock samples from Fault Cave were also used for geochemical analyses. Unaltered embedded bedrocks, either dolomites (two samples at Sterkfontein Cave) and cherts (one sample at Sterkfontein Cave and three at Fault Cave) collected during previous field campaigns, were also selected as primary, intact reference lithologies for geochemical analyses.

### Geochemical analyses

Filtered groundwater samples from each cave were diluted using 1 M HNO_3_ to obtain three final aliquots per cave at 0.5 M HNO_3_, similarly to blanks and standards used for quantitative-inductively coupled plasma mass spectrometry (Q-ICP-MS) analyses.

Selected samples of embedded pristine dolomites and cherts, and ghost-rocks were finely crushed using a clean agate mortar. As most samples contained both carbonate and silicate fractions in variable proportions, 200 mg of each sample were used for sequential leaching. The carbonate phase was first digested in clean Teflon^®^ vials using 0.5 M hydrochloric acid (HCl). After 10 min of sonication and overnight equilibration, solutions were evaporated to dryness during 24 h at 85°C. Solid residues were rinsed twice with Milli-Q^®^ water, and the supernatants were recovered after centrifugation to obtain the carbonate leach for each sample. The remaining solid residues were evaporated to dryness overnight at 75°C. These residues, along with chert samples, were then digested in Teflon^®^ vials using 1 ml of 27 M hydrofluoric acid (HF), 0.6 ml of 16 M HNO_3_ and 300 µl of Milli-Q^®^ water. Samples were left 24 h at 110°C, then evaporated to dryness overnight at 110°C. To neutralize fluorides and oxides, sample residues were treated with either aqua regia (i.e. concentrated HNO₃ and HCl mixture) or 12 M HCl during 24 h to 48 h at 125°C or 110°C, respectively. After complete digestion, samples were evaporated to dryness, then diluted with 16 M NHO_3_ and heated at 120°C during 24–48 h.

All groundwater and solid samples were analyzed using Q-ICP-MS on an Agilent 7900 at the IPGP PARI platform (Paris, France) using standard bracketing method with SLRS-6 standard (NRC of Canada) for groundwater and USGS BCR-2 reference material for solid samples. Reproducibility on standards was commonly better than 10% for groundwater and 3% for solids. Blank analyses (HNO_3_ 0.5 M) were performed after every eight samples. Results are detailed in Supplementary Table S2.

Total organic carbon (TOC) analyses were conducted at the Service d’analyse des roches et des minéraux (SARM, CRPG, Nancy, France) using 250 mg of each solid sample that were decarbonated using water and 2% HCl, then filtered to remove chlorides. TOC was measured with a HORIBA EMIA-320V2 carbon/sulfur analyzer via infrared light absorption during combustion in an oxygen flow.

### DNA extraction and sequencing

DNA extractions for both groundwater and ghost-rock samples were performed at IPGP (Paris, France). For groundwater samples, DNA was extracted from both the 3 µm and 0.2 µm filters collected at each of the three sampled caves (for a total of six samples; Supplementary Table S1) using the DNeasy^®^ PowerWater^®^ Kit (QIAGEN), following the manufacturer’s protocol. For ghost-rock samples (two samples for Sterkfontein Cave, one sample for Fault Cave and one sample for Glover’s cave, with four extraction replicates for each sample) DNA was extracted from 10 g sample aliquots using the DNeasy^®^ PowerMax^®^ Soil Kit (QIAGEN), following the manufacturer’s protocol.

Extracted DNA concentrations were quantified using a Qubit™ 2.0 fluorometer with the Qubit™ dsDNA High Sensitivity Assay Kit (Thermo Fisher Scientific), and purity was assessed with a NanoDrop™ spectrophotometer (Thermo Fisher Scientific). All extraction blanks were below detection limit (<0.005 ng/µl). Sample’s extractions below detection limits were discarded. Only the samples with measurable concentrations were kept for subsequent pooling and concentration using a SpeedVac™ system (Thermo Fisher Scientific) to obtain sufficient DNA for shotgun metagenomic sequencing. DNA concentrations before pooling and concentration ranged from 0.018 to 0.140 ng/µl for ghost-rock samples and from 0.538 ng/µl to 8.200 ng/µl for groundwater. The Glover Cave ghost-rock sample and the 3 µm filter from Fault Cave yielded insufficient DNA and were thus excluded from downstream analysis. The remaining samples were sent to Eurofins Genomics™ for sequencing on an Illumina NovaSeq platform (2 × 150 base pair, bp). Read number per sample are listed in Supplementary Table S1. Nonpareil3 was used for the diversity and coverage estimates (Rodriguez-R et al. 2018) and results are listed in the Supplementary Table S1. Average coverage for all samples was of 55±9%, based on a sequencing effort of 7.3±1.2 Gbp, providing a solid basis for downstream analyses. However, increasing the sequencing effort to approximately 20 Gpb, as suggested by Nonpareil3, would likely have improve the coverage. All raw sequencing reads are deposited in the NCBI Sequence Read Archive under the Bioproject PRJNA1298313.

### Metagenomic analysis

This study combined metagenomic analyses at the read, contig and MAG levels to strengthen biological interpretations. Due to DNA extraction challenges and the limited number of samples, analyses at multiple levels provided complementary perspectives. The MAG level allows an integrated view of the metabolic pathways, but captures only a fraction of the total sequences. In contrast, read and contig level analyses, enabling the detection of specific genes, offer greater representativity. Thus, even though genome-scale pathway reconstruction is not possible, they can help confirm pathway prevalence inferred from MAGs by finding differentially abundant genes.

Raw sequences were quality-trimmed using Cutadapt (Martin 2011), with the following parameters: -m 50 –max-n 4 –nextseq-trim 20. Functional profiling was performed with Humann 3.0 against the UniRef90 database, yielding 12%–47% mapped reads across samples. Pathways were regrouped according to MetaCyc definitions (Caspi et al. 2020). The total coverage depth (in reads per kilobase) was normalized to count per millions (cpm), as recommended in the Humann documentation (Beghini et al. 2021). Two co-assemblies were generated, one for groundwater samples, and one for ghost-rock samples, using MEGAHIT (Li et al. 2015) with the following parameters: –min-contig-len 1000 –presets meta-large. Taxonomic annotation of the contigs was done using the “taxonomy” tool of MMseqs2, based on the GTDB database, with default parameters (Mirdita et al. 2021). About 96.4% and 96.9% of the contigs got a taxonomic assignation at the superkingdom level at least for the groundwaters and rocks co-assemblies, respectively. Community analysis was then based on the percentage of total contig coverage. Functional annotation of the contigs was done using Prokka v1.14.5 (Seemann 2014) and KofamScan (Aramaki et al. 2020). Significant differentially abundant KEGG Orthologs (KOs) between ghost-rock and groundwater samples were identified using LEfSe (Segata et al. 2011) with default parameters. It performs a Kruskal–Wallis sum-rank test followed by linear discriminant analysis (LDA) to evaluate effect size of KEGG annotations.

Contigs coverage was calculated using Bowtie2 (Langmead and Salzberg 2012), and bining was done with MetaBat2 (Kang et al. 2019) with a minimum contig length of 1500 bp. Bins were manually refined using Anvi’o (Eren et al. 2015), dereplicated with dRep (Olm et al. 2017), quality-controlled with CheckM (Parks et al. 2015), and taxonomically classified using GTDB-Tk (Chaumeil et al. 2020). This process yielded 273 MAGs of good quality (> 50% completeness, < 5% contamination), recruiting between 18% and 62% of reads from groundwater samples, and between 29% and 45% of reads from ghost-rock samples. Among these, the 94 high-quality MAGs (> 90% completeness, < 5% contamination) were deposited in NCBI under the Bioproject PRJNA1298313. The taxonomy, completeness, contamination, NCBI accession numbers and N50 values of the 273 MAGs are listed in Supplementary Table S3. The prefix of the MAG names (South Africa Rocks, SAR or South Africa Water, SAW) differentiates those generated using the ghost-rocks co-assembly from those generated using the groundwaters co-assembly.

To estimate MAG coverage per sample types, mean contig coverage values provided by Anvi’o were normalized for each sample by its global mean contig coverage. Then, normalized MAG coverage values were averaged across the five groundwater samples, and the three ghost-rock samples.

Concerning functional gene analyses, genes related to autotrophy were identifed with hmmsearch (Eddy, 2011) using HMM profiles from Zhou et al. (2022b). Carbon fixation through the hydroxypropionate/hydroxybutyrate cycle was investigated by Basic Local Alignment Search Tool (BLAST) using the sequences from *Nitrosopumilus maritimus* (Könneke et al. 2014, Otte et al. 2015). The classification of hydrogenases was conducted using the HydDB web server (Søndergaard et al. 2016). AntiSMASH v7.0.1 (Blin et al. 2023) was used to search for secondary metabolite biosynthetic gene clusters, with the –hmmdetection-strictness parameter defined as “strict”. Carbohydrate-active enzymes (CAZymes) were identified with dbCAN3 (Zheng et al. 2023) with default parameters. The CAZymes belonging to the GH13, GH57, GH77, GT5 and GT35 families were analyzed specifically, as they are characteristic for α-glucan degradation (Colpaert et al. 2020). A MAG is defined as a glycogen synthesizer if it possesses one of the following CAZyme combinations: [GT5 or GH77 or (GH13_6 + GH13_3)] + (GH13_8 or GH13_9 or GH57), based on Colpaert et al. (2020). This CAZyme-based approach was compared to a KofamScan-based approach, where glycogen synthetizers/degraders are detected by complete Kegg modules M00854-M00855. The preponderance of glycogen degraders/synthetizers MAGs was highlighted by their percentage of the total MAGs coverage.

MAG phylogenomic analysis was done using 40 marker genes extracted with fetchMG v1.2 (https://github.com/motu-tool/fetchMGs), aligned with Multiple Alignment using Fast Fourier Transform (MAFFT) using default parameters (Katoh et al. 2002), and concatenated with catfasta2phyml.pl (https://github.com/nylander/catfasta2phyml). Phylogenetic reconstruction was performed with IQ-TREE (Minh et al. 2020) with ModelFinder (Kalyaanamoorthy et al. 2017) to select the best-fit model (LG+F+R10), and statistical support was assessed with 1000 ultrafast bootstrap (Hoang et al. 2018) and 1000 SH-like approximate likelihood ratio test replicates (Guindon et al. 2010). The alignment contained 16 969 parsimony-informative sites. The resulting tree was colored according to GTDB-Tk taxonomic assignments.

### Search for metal-cycling metabolic pathways

Considering the putative importance of metal redox cycling in the Sterkfontein karst system, several classes of metal-interacting pathways were systematically screened in reconstructed MAGs.

First, micro-organisms are known to synthesize metallophores, which as specialized chelators for nutrient acquisition through metal chelation and mobilization (Kraemer et al. 2015). A metallophore BGC is a clustered group of genes that together encode a biosynthetic pathway for the production of a metallophore. They were searched with antiSMASH v7, which incorporates updated detection rules for additional metallophore clusters (Blin et al. 2023).

Second, MCOs are a type of oxidase that use Cu cofactors for the oxidation of a wide range of substrates, transferring the electrons to O_2_. An Mn-oxidizing activity has been described in a few MCOs (Zhou and Fu 2020). They were searched using Prokka functional annotations.

Third, EET systems allow micro-organisms to use metals as terminal electron donors or acceptors (Shi et al. 2016). Most of the known EET systems typically involve multiple *c*-type cytochromes, in particular multiheme cytochromes (MHCs) (Shi et al. 2016). MHCs are often found in porin-cytochrome complexes (PCCs) (Hartshorne et al. 2009, Richardson et al. 2012, Shi et al. 2014, He et al. 2017), which have been described in numerous metal oxidizing or reducing micro-organisms (Szeinbaum et al. 2020, Eddie et al. 2022, Yu et al. 2022), including electroactive organisms from sediments and groundwaters (Arbour et al. 2020). Furthermore, most PCCs in both metal-oxidizers and -reducers are centered on at least one MHC with ≥ 10 heme-binding motifs (Shi et al. 2016). The presence of such MHCs has been proposed either to predict microbial iron reduction capacity (Hernsdorf et al. 2017) or to help distinguish iron-oxidizing bacteria from nitrite-oxidizers among the Gallionellaceae (Hoover et al. 2023). Based on these insights, the search for PCCs in MAGs was done using three criteria, (i) the presence of one MHC with at least 10 heme-binding sites, (ii) the presence of at least one additional *c-*type cytochrome in close genomic proximity and (iii) the presence of one porin predicted to contain at least 10 β-strand transmembrane domains.

*C-*type cytochromes were detected using the heme_counter.pl script (https://github.com/seanmcallister/heme_counter), modified to find any CX_2_-_4_CH motif (since variants to the canonical CXXCH motif are known; Sharma et al. 2010, Edwards et al. 2020). PRED-TMBB2 (using the MSA-version and HMM option) was used to search for putative porins (Tsirigos et al. 2016). Cellular localization of each PCC components was inferred with PSORTb v3.0.3 was used (Yu et al. 2010). All identified MHCs were clustered using MMseqs2 (Steinegger and Söding 2017) with default parameters, corresponding to a minimum sequence identity of 90%. Genomic context and gene cluster illustrations were generated using the Python package pyGenomeViz (https://github.com/moshi4/pyGenomeViz) (Gilchrist and Chooi 2021).

As Cyc2 is a widely used protein for iron oxidation (McAllister et al. 2020, Zhou et al. 2022a), FeGenie (Garber et al. 2020) was used with default parameters for their characterization in the MAGs.

## Results

### Geochemical analyses

The geochemical composition of groundwater samples (Supplementary Table S2) reflected the dominant lithology of the dolomitic bedrock, with the predominance of Ca and Mg (53.5–93.7 mg/l and 32.8–55.1 mg/l for Ca and Mg respectively). Notably, Fault Cave and Sterkfontein Cave samples presented very similar concentrations, consistent with the hydrological connection between the two caves.

**Figure 2:**
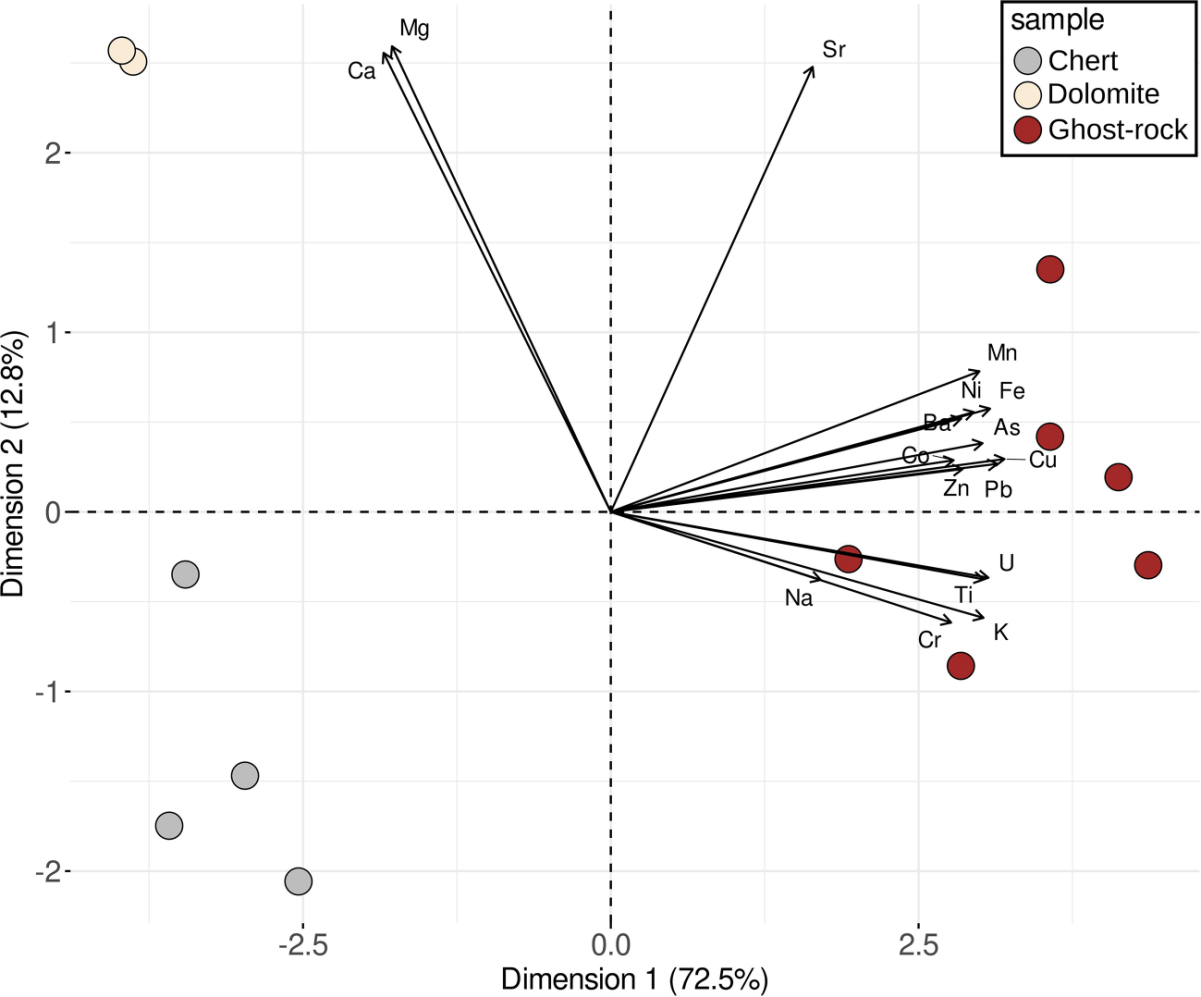
Principal component analysis (PCA) of embedded chert, dolomite, and ghost-rock samples based on major and trace element concentrations. The samples of embedded chert and dolomite are in grey and beige respectively, and the ghost-rock samples are in brown.

Major and trace elemental concentrations of rock samples, either ghost-rocks or embedding rocks (Supplementary Table S2) were further analyzed with principal component analysis (PCA; Fig. 2), with the first two PCA dimensions accounting for 85.3% of the total variance. These dimensions clearly differentiate dolomite, chert and ghost-rock samples, consistent with their dominant mineralogy.

PCA dimension 2 explained 12.8% of the variance, primarily controlled by Ca and Mg, with contributions from Sr, which clearly separated dolomite samples from ghost-rocks and cherts. Dolomite samples exhibited particularly high Mg and Ca concentrations (11.2 wt% and 19.6±0.7 wt%, respectively) compared to cherts [(1.3 ± 1.6) × 10^4^ µg/g and (2.2±2.8) × 10^4^ µg/g, respectively] and ghost-rocks [(7.9±2.7) × 10^3^ µg/g and (8.9±4.7) × 10^3^ µg/g, respectively]. The lower Ca and Mg concentrations in ghost-rocks and chert confirmed that secondary calcite or dolomite were not dominant in these samples, in contrast to secondary metal oxides (Pisapia et al. 2021). This was supported by data dispersion on dimension 1 (which explains 72.5% of the variance) that reflected variations in trace metals, particularly iron and manganese, clearly distinguishing ghost-rocks from unaltered lithologies. Ghost-rocks were enriched in Fe and Mn [(2.0±0.6) × 10^4^ µg/g and (2.6±0.9) × 10^4^ µg/g, respectively] compared to dolomites (2030±21 µg/g and 2460±99 µg/g, respectively) and cherts (782±942 µg/g and 406±484 µg/g, respectively). This enrichment was consistent with the presence of Fe and Mn oxides in ghost-rocks (Pisapia et al. 2021). Ghost-rock samples were also enriched in heavy metals such as As, Pb, U, Ni, Zn, Ti, and Cu compared to dolomite and cherts (Supplementary Table S2). Overall, these result indicated that ghost-rocks were enriched in redox sensitive elements, such as iron and manganese, which may be bioavailable for chemolithotrophs.

Even though organic matter has previously been observed in ghost-rocks (Pisapia et al. 2021), and some DNA extractions yielded sufficient material for sequencing, the TOC content in all dolomite, chert and ghost-rocks samples were below the detection limit of 0.15 wt%, which confirmed that karstic environments, including ghost-rocks, can be considered as highly oligotrophic environments.

### Taxonomic and functional description of the microbial communities at the contig level

**Figure 3:**
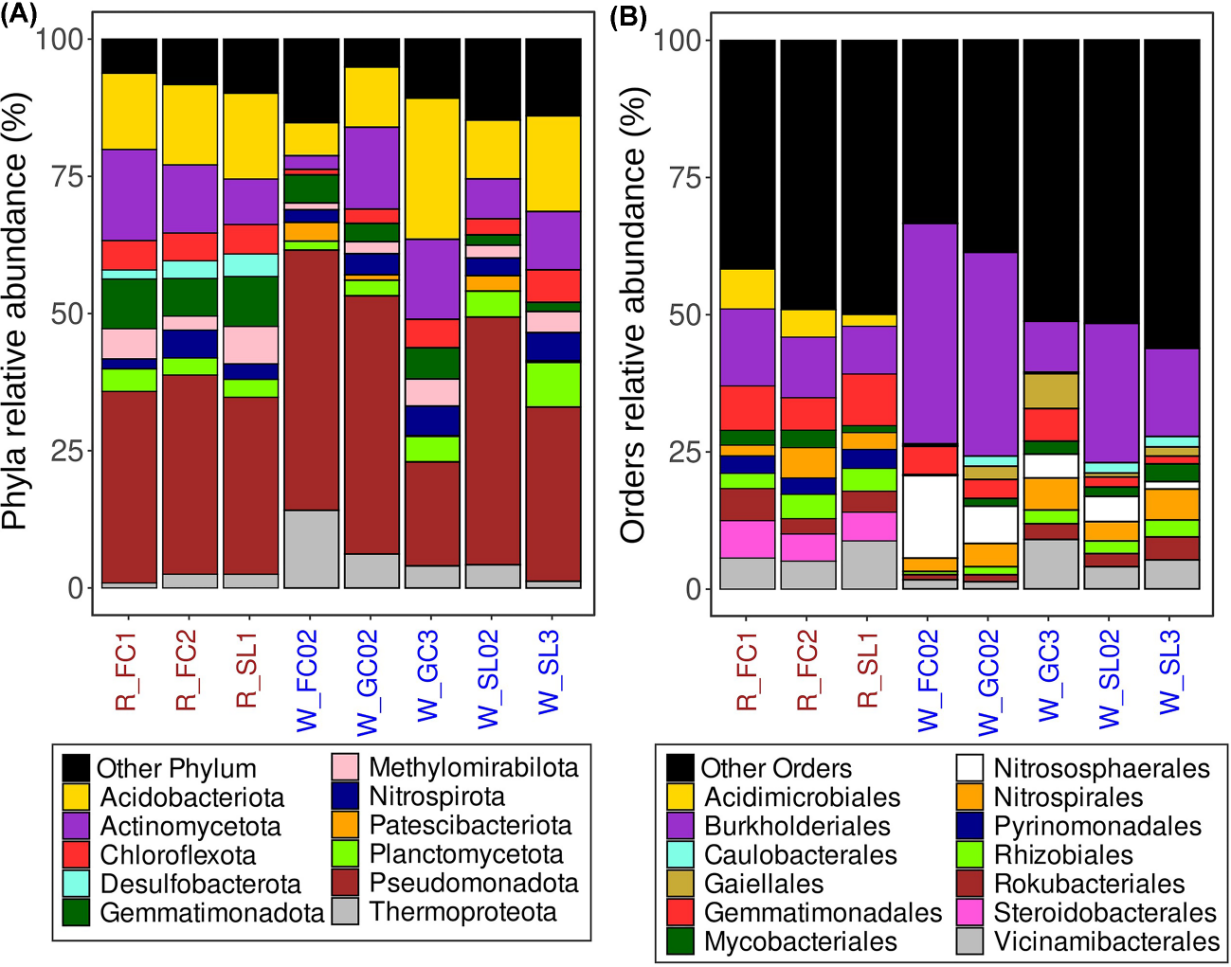
Microbial community at the contig level. (A) Phyla abundance based on the percentage of the total contig coverage. (B) Orders abundance based on the percentage of the total contig coverage.

In groundwater and ghost-rock samples, the most abundant phyla were Pseudomonadota (38.1±12.5% and 34.5±2.0%, respectively), Acidobacteriota (14.2±7.6% and 14.7±0.9%, respectively), Actinobacteriota (10.0±5.2% and 12.4±4.2, respectively), Gemmatimonadota (3.5±1.9% and 8.3±1.3%, respectively), Thermoproteota (6.0±5.0% and 2.0±0.9%, respectively), Chloroflexota (3.5±2.0% and 5.3±0.2%, respectively), Methylomirabilota (2.9±1.5% and 5.0±2.2%, respectively), Planctomycetota (4.4±2.5% and 3.5±0.6, respectively), and Nitrospirota (4.0±1.3% and 3.2±1.7%, respectively) (Fig. 3A). The orders that were relatively more abundant in groundwaters compared to ghost-rocks were the Burkholderiales (36±30%), the Nitrososphaerales (5.4±5.3%), the Nitrospirales (3.6±2.2%) (Fig. 3B).The orders that were relatively more abundant in ghost-rocks compared to the groundwaters arewere the Gemmatimonadales (7.8±1.8%), the Vicinamibacterales (6.5±2.0%), the Steroidobacterales (5.7±1.0%), the Acidimicrobiales (4.8±2.6%), the Rokubacteriales (4.13±1.6%), the Rhizobiales (3.8±0.9%), and the Pyrinomonadales (3.2±0.2%) (Fig. 3B).

In order to highlight the functional differences between ghost-rock and groundwater samples, a LEfSe analysis at the contig level was used to identify differentially abundant KOs and to pinpoint those that best discriminate groundwater from ghost-rock samples. In total, 403 annotations had a LDA score greater than 2, with 192 in groundwaters and 211 in ghost-rocks. They were grouped by KEGG pathways; 89 Kos were not assigned any KEGG pathway. All KEGG pathways with significant KOs are listed in Table 1. The complete list of significant KO annotations is provided in Supplementary Table S4.

**Table 1:** LEfSe analysis of KEGG annotations from all the contigs.

Kegg pathway	Ghost-rocks	Groundwater
00 362 Benzoate degradation	0	14
00 440 Phosphonate and phosphinate metabolism	0	5
00 907 Pinene, camphor and geraniol degradation	0	4
02 035 Bacterial motility proteins	0	7
03 036 Chromosome and associated proteins	0	4
03 400 DNA repair and recombination proteins	0	4
00 500 Starch and sucrose metabolism	4	0
09 109 Metabolism of terpenoids and polyketides	17	0
00 260 Glycine, serine and threonine metabolism	4	3
00 330 Arginine and proline metabolism	5	2
01 002 Peptidases and inhibitors	7	4
02 000 Transporters	31	26
02 020 Two-component system	4	11
02 044 Secretion system	2	8
03 000 Transcription factors	12	20
03 009 Ribosome biogenesis	2	4
09 108 Metabolism of cofactors and vitamins	7	2
Other categories (<4 KOs)	60	41

A total of 403 significant KEGG annotations was obtained and grouped according to KEGG pathway definitions. Categories containing less than four significant KEGG annotations in both groundwater and ghost-rocks are labeled as “Other categories” and are not discussed further. Categories enriched in groundwater and ghost-rocks are shown at the top and in the middle, respectively. Categories with KOs enriched in both groundwater and ghost-rock are at the bottom.

Most significantly enriched KOs fell into broad functional categories essential for general cellular processes (notably transporters with 57 KOs and transcriptions factors with 32 KOs), as well as other generic functional groups (shown in black in Table 1). Such categories were expected to include KOs enriched in both groundwater and ghost-rocks. In contrast, several more specific functional categories contained KOs enriched exclusively in groundwater or in ghost-rocks.

Among functions enriched only in ghost-rocks, 17 KOs were involved in terpenoid and polyketides biosynthesis and 4 KOs related to starch and sucrose metabolism. Terpenoid and polyketide KOs were mostly involved in type II polyketide biosynthesis (K05551, K05552, K05554, K12420, K13327, K14631, K15926, K15928) and in carotenoid biosynthesis (K06443, K10211, K10212, K15746). The starch/sucrose-related KOs include key CAZymes involved in α-glucan catabolism (K01176: a-amylase [EC:3.2.1.1], K01187: α-glucosidase [EC:3.2.1.20], K01194: α,α-trehalase [EC:3.2.1.28], K02438: glycogen debranching enzyme [EC:3.2.1.196]). Two enriched transporter KOs (K10232: α-glucoside transport system substrate-binding protein, K21572: starch-binding outer membrane protein, SusD/RagB family) further suggest enhanced α-glucan uptake in ghost-rock communities.

Genes involved in aromatic compound degradation were strongly enriched in groundwaters. They included 14 KOs involved in benzoate degradation, such as K00446 encoding catechol 2,3-dioxygenase [EC:1.13.11.2], and K16242-K16249, which correspond to the phenol/toluene 2-monooxygenase (NADH) operon [EC:1.14.13.244;1.14.13.243]. Two KOs related to aminobenzoate degradation were also significantly enriched (K03862 and K15064). They correspond to the vanillate monooxygenase [EC:1.14.13.82] and the syringate O-demethylase [EC:2.1.1.-], respectively. Additional groundwater-specific KOs belonged to monoterpene (pinene, camphor, and geraniol) degradation (K11731, K13774, K13777, K13778), phosphonate degradation (phnGHIJ), DNA repair and chromosomal proteins, as well as motility/chemotaxis (K03415, K03776), and multiple flagellar proteins and regulators (K02423, K06603, K02398, K02402, K02403).

Overall, the contig-level analysis highlighted a clear enrichment of α-glucan degradation functions in ghost-rock and aromatic compound degradation in groundwater samples.

### Metabolic specificities of groundwater and ghost-rock communities at the MAG level

#### Groundwater samples

To confirm and expand the results obtained at the contig level, the 273 reconstructed MAGs were analyzed. In groundwater samples, the community was dominated by members of the Gammaproteobacteria, Nitrospirota, and Thermoproteota phyla, together accounting for 54% of total MAG coverage, which was consistent with the results at the contig level. This dominance, however, was strongly influenced by a single gammaproteobacterial MAG (SAR200) assigned to *Limnobacter* sp. This MAG harbored pathways involved in benzene degradation (with the phenol/toluene 2-monooxygenase; K16242-K16246, K16249) and the catechol meta-cleavage pathway (with the catechol 2,3 dioxygenase; K00446), as well as the complete *soxABCDXYZ* complex for thiosulfate oxidation (K17222-K17227, K22622). Because no carbon fixation pathway was detected, SAR200 was considered as a chemolithoheterotroph.

Overall, chemolithoheterotrophic and chemolithoautotrophic MAGs represented almost half of total MAG coverage in groundwater samples (47.6%). Ten of the 17 MAGs belonging to the Nitrososphaerales order in the Thermoproteota phylum possessed the genes needed for carbon fixation through the 3-hydroxypropionate/4-hydroxybutyrate cycle. The most abundant Thermoproteota MAG (SAW134) contained homologues for all the enzymes demonstrated or proposed for *Nitrosopumilus maritimus* in the pathway with BLASTp identities between 77% and 91%. SAW134 also possessed genes for an ammonia transporter (K03320), the *ureABCDEFG* operon (K01428-K01430, K03187-K03190), and the ammonia monooxygenase complex *amoABC*, (K10944-K10946). Twelve MAGs within the Nitrospirota phylum possessed at least one subunit of the ATP-citrate lyase (K15230-K15231), a key enzyme for the reverse tricarboxylic acid (TCA) carbon fixation pathway. Among them, four MAGs also had genes for the nitrite oxidoreductase complex (K00370-K00371) needed for nitrite oxidation.

Other abundant autotrophic MAGs in groundwater possessed the genes encoding ribulose-1,5-bisphosphate carboxylase/oxygenase (RuBisCO; K01601-K01602) and the Calvin-Benson-Bassham (CBB) cycle for carbon fixation, and belonged to diverse Gammaproteobacteria genera, including *Methylibium* (SAW204, SAW232, SAW19), *Panacagrimonas* (SAW210, SAW216), *Aquabacterium* (SAW306, SAW104), and *Polaromonas* (SAW79, SAR163). Three of these had the genes encoding the complete *soxABCDXYZ* complex for thiosulfate oxidation (SAW79, SAR163, and SAW210), while the two *Aquabacterium* MAGs encodeed a sulfide: quinone oxidoreductase (*sqr;* K17218) for sulfide oxidation. In addition, three MAGs (SAW104, SAW216 and SAW210) may be metal oxidizers as they also possessed genes encoding a PCC (see next Section).

Among chemoorganoheterotrophic taxa, the SAR211 MAG, assigned to *Acidovorax delafieldii*, was the most abundant in groundwater samples. It possessed a complete glycolysis pathway and TCA cycle, as well as genes for dissimilatory nitrate reduction to ammonia (*nirBD;* K00362-K00363; *narGHI;* K00370-K00371, K00374) and pathways for benzoate, benzene and salicylate degradation.

As the analyses at the contig level showed the prevalence of pathways involved in aromatics and carboxylate degradation in groundwaters, their distribution was further verified at the MAG level. Only 15 MAGs possessed at least one complete KEGG module for aromatic compound degradation (SAR163, SAR200, SAR211, SAW152, SAW160, SAW19, SAW204, SAW210, SAW216, SAW232, SAW267, SAW277, SAW309, SAW315, SAW77). They represented 35.7% of total MAG coverage in groundwater but only and 0.4% in ghost-rock. At the read level, pathways involved in aromatics degradation were also significantly relatively more abundant in groundwater samples (mean = 27.4 cpm) than in ghost-rock samples (mean = 4.0 cpm; p = 0.03571), especially those targeting catechol and protocatechuate degradation (Supplementary Fig. S1).

Carboxylates offers another source of organic matter for groundwater heterotrophs, as highlighted by read level-analyses, where pathways involved in carboxylate degradation were significantly relatively more abundant in groundwater samples (mean = 81.8 cpm) than in ghost-rock samples (mean = 5.7 cpm; p = 0.03571; Wilcoxon exact test) with D-galactarate and D-glucarate degradation being particularly enriched (Supplementary Fig. S3). Accordingly, 11 MAGs had genes for D-glucarate and/or D-galactarate dehydratase (Supplementary Fig. S2), including 7 MAGs belonging to the Burkholderiales order (*Polaromonas* SAW152, SAR163, SAW79; *Acidovorax* SAR211; *Rubrivivax* SAW179; *Methylibium* SAW204; and SAW317, unassigned Burkholderiales). They represented 8.3% and 1.9% of total MAGs coverage in groundwater and ghost-rock samples, respectively, confirming the enrichment of these pathways in groundwaters communities. The genomic context of these genes was very similar to the recently described operon needed for hexuronate catabolism in Pseudomonadota (Bouvier et al. 2018), including the presence of genes encoding the uronate dehydrogenase Udh and the uronate lactonase UxuL that convert galacturonate and glucoronate into galactarate and glucarate, respectively (Supplementary Fig. S2).

#### Ghost-rock samples

**Figure 4:**
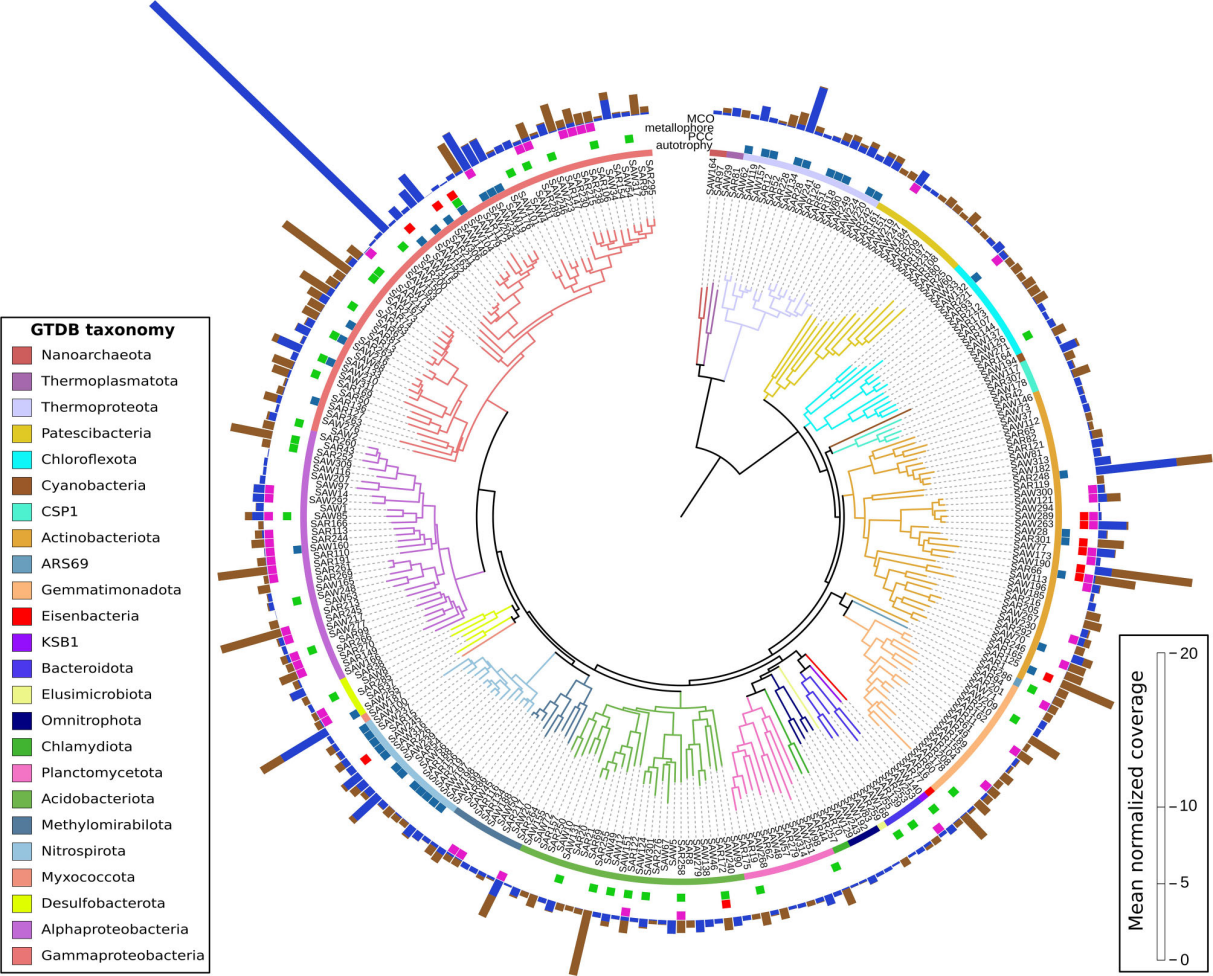
Phylogeny of the 273 MAGs obtained on the groundwater and ghost-rock samples. The branches are colored according to the taxonomy assigned by GTDB-Tk. The outer ring shows a histogram of mean normalized coverage of the MAG in groundwater samples (blue) and ghost-rock samples (brown). The four inner rings indicate MAGs harboring at least one of the following feature: multicopper oxidases (MCOs, pink square), metallophore biosynthesis pathway (red square), porin-cytochrome complexes (PCCs, green square) or carbon fixation pathway (blue square).

The predominant MAGs in ghost-rock samples belonged to the Actinobacteriota, Gemmatimonadota, Acidobacteriota, and Alphaproteobacteria, accounting for 51% of total MAG coverage (Fig. 4), which was consistent with the results at the contig level.

Chemolithoautotrophic MAGs account for 16.2% of total MAG coverage. Among them, six actinobacterial MAGs (SAR125, Acidimicrobiales; SAR66, Pseudonocardiaceae; SAR301, *Actinokineospora* sp.; SAR248, UBA4738; SAW70, Acidimicrobiales; SAW28, *Pseudonocardia*) encoded the RubisCO and a complete CBB cycle. Moreover, all six possessed genes for high-affinity [NiFe]-hydrogenase 1 h, and four possess the *coxMLS* operon involved in CO oxidation.

Two additional autotrophic gammaproteobacterial MAGs (SAR13; Steroidobacteraceae and SAR127; genus CAIKBL01), also possessed genes encoding RubisCO and the CBB cycle. SAR13 possessed, on one contig, a complete set of genes for the initial step of methanol oxidation (methanol dehydrogenase *xoxF;* K23995, glutathione-dependent formaldehyde-activating enzyme K03396; S-(hydroxymethyl)glutathione dehydrogenase K00121, and formate dehydrogenase K15022), and on another contig, genes encoding S-formylglutathione hydrolase (K01070) necessary to complete the glutathione-dependent pathway, enabling full oxidation of methanol to CO_2_. SAR127 possessed several *coxMLS* genes necessary for CO oxidation, and *soxACXYZ* genes involved in thiosulfate oxidation (*soxB* is missing).

**Figure 5:**
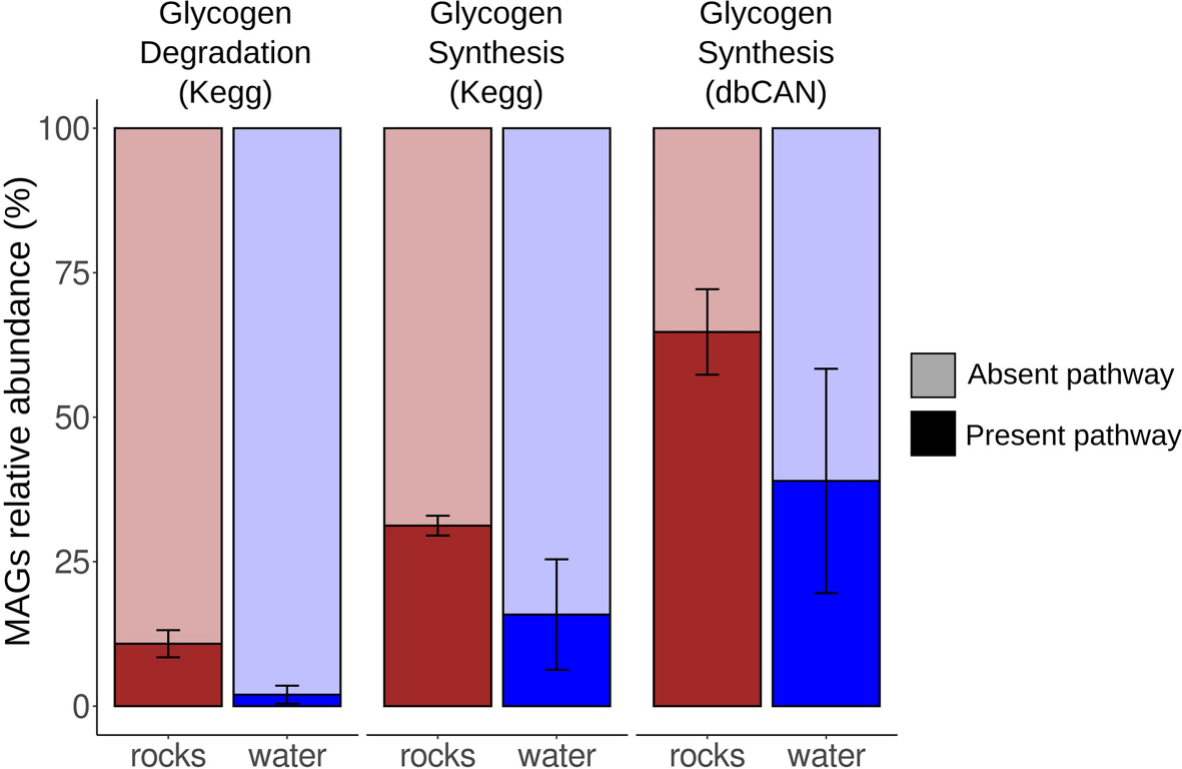
Relative abundance of the glycogen metabolism in the reconstructed MAGs. From left to right, abundance of the glycogen degraders and synthetizers based on KofamScan search, and glycogen synthetizers based on the dbCAN search. The MAG abundance is calculated as the percentage of total MAGs coverage. Ghost-rock samples are in brown and groundwater samples are in blue.

As the marker gene analysis at the contig level indicated a greater relative abundance of α-glucans (glycogen and starch) degradation pathway in ghost-rock samples, the presence of genes encoding CAZymes involved in this process within the MAGs was investigated. Based on the KofamScan annotations, the MAGs with a complete glycogen degradation or synthesis pathway represented 10.8±2.4% and 31.2±1.7% of the ghost-rock total MAGs coverage respectively, whereas in groundwaters they represented 2.0±1.6% and 15.8±9.6% of the total MAGs coverage respectively (Fig. 5). Based on the dbCAN3 annotations, the glycogen synthesizers were also relatively more abundant in ghost-rock samples (64.7±7.4% compared to 39.0±19.4% in groundwaters, Fig. 5). Overall, the glycogen metabolism represented a higher proportion of the MAGs relative abundance in ghost-rocks samples than in groundwater samples. Concerning the read level, no significant difference was detected between groundwater and ghost-rock samples in the abundance of polysaccharide degradation (mean = 24.8 and 37.0 respectively; p = 0.1429), mostly due to the lack of differential abundance of chitin degradation pathways (p = 0.37 and p = 1, for chitin deacetylation and chitin degradation II, respectively). When restricting the analysis to α-glucan degradation pathways (i.e. pathways involved in glycogen and starch degradation), a significant difference emerged, with a higher relative abundance of these pathways in ghost-rock samples (mean = 28.4 cpm) compared to groundwater samples (mean = 14.4 cpm; p = 0.03571) (Supplementary Fig. S1C).

As the LEfSe analysis also showed that the production of certain secondary metabolites was characteristic of ghost-rock communities, the metabolic potential for secondary metabolites biosynthesis was further inspected across the reconstructed 273 MAGs. In total, 715 BGCs have been found, with up to 35 BGCs found in a single MAG. Most of these clusters showed no or low similarity to any entry in the minimum information about a biosynthetic gene cluster (MIBiG) database (Supplementary Fig. S3), suggesting the presence of novel or uncharacterized secondary metabolites. The majority of BGCs were involved in terpene (222 clusters), polyketide (96 clusters), and non-ribosomal peptide (41 clusters) biosynthetic pathways.

No significant difference was observed in the mean number of BGC per MAG between groundwater (mean = 1.64) and ghost-rock (mean = 2.07) samples (p = 0.95). Nevertheless, a slight but significant positive correlation existed between the mean coverage of MAGs in ghost-rock samples and their number of BGCs (Spearman’s r = 0.15, p = 0.012). It was mainly due to the high abundance of three Actinobacteriota MAGs in ghost-rock samples: SAR301 (assigned to the genus *Actinokineospora*), SAR66 (Pseudonocardiaceae) and SAW190 (Pseudonocardiaceae, genus GCA-003244245), which contained 35, 30 and 19 BGCs, respectively (Supplementary Fig. S4).

127 MAGs possessed a putative O_2_ reducing terminal oxidase (based on the presence of a complete Kegg module M00153-M00156 or M00416-M00417), which was expected as both groundwaters and sampled ghost-rocks were in contact with the atmosphere and oxygenated.

### Analysis of the metal-interacting pathways

As ghost-rock samples are enriched in redox-sensitive elements, notably Fe and Mn (Fig. 2), reflecting active metal cycling and potential (bio)alteration/(bio)mineralization processes, a specific attention has been paid to genes and pathways involved in metal interactions, including metallophores, MCOs and PCC synthesis (Fig. 4).

Metallophore-producing BGCs were found in 12 MAGs, and 45 MAGs encoded at least one MCO, primarily among Alphaproteobacteria and Actinobacteriota, in both groundwater and ghost-rock samples. Concerning EET systems, 25 MAGs possessed at least one Cyc2 encoding gene, but all these genes were assigned to the cluster 3, implying that their role in iron oxidation should be considered uncertain (Keffer et al. 2021). Thirty six MAGs harbored at least one PCC, of which 29 have at least 10 multiheme cytochromes (Supplementary Table S2) and 27 also possessed *ccm* genes required for cytochrome *c-*type biogenesis. Considering the fragmented nature of the MAGs, we estimate that at least 75% of the MAGs possessing a PCC also encoded a functional cytochrome c biogenesis pathway. PCCs were not evenly distributed among phyla: they were all in groups of bacteria with an outer membrane, except for SAW271, assigned to the Chloroflexota phylum, and SAR286 and SAW140, which belonged to uncultivated candidate phyla (ARS69 and KSB1, respectively). However, the presence of PCCs in KSB1 MAGs had previously been reported (Suarez et al. 2023), along with Planctomycetota MAGs that also encoded PCCs. Among the 36 PCC-containing MAGs, 21 belonged to the Gammaproteobacteria class or Acidobacteriota phylum, and two to the Planctomycetota phylum. No clear difference was observed in PCC abundance between groundwater and ghost-rock samples. PCC containing-MAGs represented 28% of total MAG coverage in groundwater, mainly due to SAR200, which dominated this community, and only 17% in ghost-rocks. Only three PCC-containing MAGs also possessed a carbon fixation pathway (Fig. 4), suggesting that most MAGs corresponded to heterotrophic bacteria that may use their PCC systems to export electrons extracellularly.

**Figure 6:**
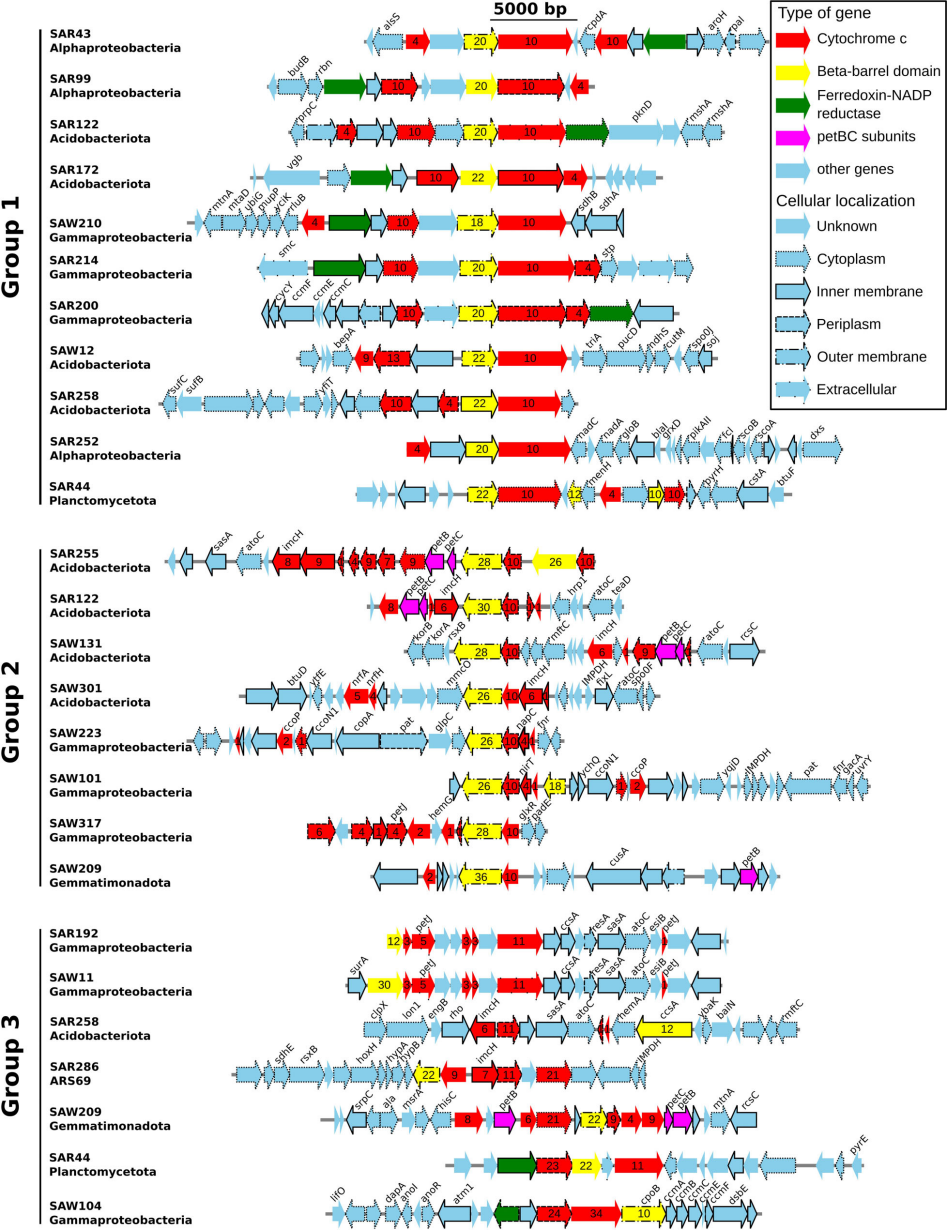
Genomic organization of extracellular electron transfer (EET) complexes detected in the MAGs. The phylum or class of the MAGs possessing the porin-cytochrome complexes (PCC) is indicated on the left. Arrows length is proportional to the length of the genes. Color indicates gene types: c-type cytochromes (red), porins (yellow), ferredoxin-NADP reductases (green), cytochrome bc_1_ subunits (pink) or other genes (light blue). For c-type cytochromes and porins, the number inside the arrow corresponds to the number of heme-binding motifs and transmembrane β-barrel domains, respectively. Arrow borders denote predicted protein cellular localization (PSORTb), and functional annotations from Prokka are indicated above each arrow.

The clustering of the multiheme cytochromes revealed several groups of PCCs sharing conserved genomic and structural features (Fig. 6). The first group comprising 11 PCCs was characterized by the presence of a large decaheme cytochrome (between 4 and 5 kbp) located adjacent to an outer membrane porin with around 20 transmembrane β-strands. In seven of these eleven PPCs, a ferredoxin-NADP^+^ reductase gene was also found in close genomic proximity, suggesting potential functional coupling. The second group, which included 8 PCCs had smaller decaheme cytochromes located next to an outer membrane porin containing between 25 and 36 transmembrane β-strands. Six of them also encoded inner-membrane cytochromes similar to ImcH or NapC/NirT that were necessary for electron transfer in iron oxidizers or reducers as they are known to interact with the quinone pool (Jain et al. 2022, Pimenta et al. 2023). The third group comprised 7 PCCs centered around undecaheme cytochromes or multiheme cytochromes with more than 20 CX_2_-_4_CH heme-binding motifs. Undecaheme cytochromes have been described in iron-oxidizing Tenderiales (Eddie et al. 2022) and in the manganese-reducing bacterium *Candidatus Dechloromonas occultata* (Szeinbaum et al. 2020). Finally, several PPCs contained the *petB* and *petC* genes, encoding the cytochrome b and Fe-S subunits of the cytochrome bc_1_ complex, respectively (SAR255, SAR122, SAW131, SAW209 in group 2, and SAW209 in group 3). The co-occurrence of these redox-active components was also described in “PCC4” systems reported by He et al. (2017), suggesting that similar electron transfer architectures may be conserved across phylogenetically diverse bacteria.

## Discussion

### Structure of the groundwater and ghost-rock microbial communities

Results based on both contig and MAG level revealed distinct communities inhabiting ghost-rocks (dominated by Actinobacteriota, Gemmatimonadota, Acidobacteriota, and Alphaproteobacteria) and groundwaters (enriched in Gammaproteobacteria, Nitrospirota, and Thermoproteota). This result was consistent with other karstic cave metabarcoding studies (Turrini et al. 2024).

While this is the first characterization of the metabolic potential of ghost-rock ecosystem, karstic groundwater communities have been more extensively studied. The groundwaters assemblage described here may represent the “core microbiome” of undisturbed groundwater, as described in Geesink et al. (2022). This community is described in this article as sustained by autotrophic and mixotrophic taxa that primarily use surface-derived inorganic nitrogen and rock-derived inorganic sulfur compounds, as well as heterotrophs capable of degrading refractory surface-derived plant biomass.

Concerning carbon sources, comparative analysis at the read, contig and MAG levels showed a preponderance in groundwater samples of aromatic compounds’ catabolic pathways, involving protocatechuate, catechol, syringic acid, vanillic acid that may derive from lignin depolymerization, and funneling pathways (Brink et al. 2019). At both the read and MAG level (but not at the contig level), D-galactarate and D-glucarate degradation pathways, both linked to the breakdown of pectin and hemicellulose (Bouvier et al. 2018), were also more abundant in groundwaters. Overall, these results indicate that refractory, surface-derived plant compounds were an important carbon source for groundwater community. In contrast, at the read, contig and MAG levels, ghost-rock communities displayed a preponderance of pathways involved in metabolism of α-glucans, notably glycogen. Ghost-rocks sampled just above the water table experience intermittent flooding and desiccation along irregular supply of both allochthonous and autochthonous organic compounds, which would induce both oligotrophic and hydric stress. Glycogen and more generally α-glucan function as major and durable carbon and energy storage molecules that enable prokaryotes to overcome environmental stresses such as desiccation and nutrient limitation (Colpaert et al. 2020, Wang et al. 2020). The ability to synthesize glycogen-like molecules likely provide a competitive advantage supporting survival during nutrient scarcity for the micro-organisms inhabiting the ghost-rocks. Glycogen may also serve as a major carbon source for heterotrophic necromass degraders in ghost-rock community. A process of α-glucan necromass turnover has previously been described in marine ecosystems, where it participates in intra-population energy conservation mechanisms (Beidler et al. 2024).

Concerning energy sources for chemolithotrophs, electron donors mobilized by the groundwater community were found to be thiosulfate (*Limnobacter, Polaromonas, Panacagrimonas*), nitrate/nitrite (Nitrospirota, Thermoproteota), or metals (*Aquabacterium, Panacagrimonas*). All cultivated *Limnobacter* species, isolated from marine, freshwater or soil environments, are thiosulfate oxidizers (Kim et al. 2024), and this study is the first report of groundwater community dominated by this genus. Conversely, thiosulfate-oxidizing *Polaromonas* (Taubert et al. 2022) and ammonia-oxidizing bacteria and archaea coexisting with nitrite-oxidizing bacteria (Krüger et al. 2023) were previously described in karstic groundwaters. The putative iron-oxidizing activity of *Aquabacterium* and *Panacagrimonas* is less documented but is consistent with the isolation of an autotrophic iron-oxidizing *Aquabacterium* strain from freshwater sediments (Straub et al. 2004). In contrast, the ghost-rock primary producers were mainly found to be Actinobacteriota putatively relying on H_2_ and CO oxidation, and Gammaproteobacteria capable of methanol oxidation. Autotrophic H_2_- and CO-oxidizing Actinobacteriota have already been described in diverse environments, including soils and marine sediments (Grostern and Alvarez-Cohen 2013). This is also the case for the gammaproteobacterial Pseudomonadota that are known autotrophic methylotrophs combining methanol oxidation to CO_2_ using the glutathione-linked formaldehyde oxidation pathway, and the CBB cycle, as described for *Paracoccus denitrificans* (Chistoserdova 2011). Volatile H_2_, CO and methanol can be found as trace gases in the atmosphere (Stacheter et al. 2013, Bay et al. 2021) and their oxidation fuels a primary production process known as “atmospheric chemosynthesis”, which prevails in oligotrophic, low-light terrestrial environments such as Antarctic soils and cold deserts (Ji et al. 2017, Ray et al. 2022), and may in fact be more widespread globally (Bay et al. 2021). Ghost-rocks, sampled up to 20 cm into highly porous cave walls, are in direct contact with cave air and situated in the unsaturated karst zone, which offers surface-like geochemical conditions (Covington et al. 2023). Thus, it is hypothesized that primary production in ghost-rocks may be similar to that in low-light oligotrophic soils, relying on atmospheric trace gas oxidation rather than photosynthetic inputs (Ji et al. 2017, Ray et al. 2022).

Even though these results provided the first insights into ghost-rock microbial community composition and metabolic potential, several questions remain. The ghost-rock community is not merely a filtered subset of the groundwater microbiome. It is a distinct community that should be adapted to the specific conditions of the ghost-rock environment. However, its biogeographic origin remains uncertain. Given the strong connectivity between caves and surface systems, endemism, as suggested for some deep subsurface lineages (Colman et al. 2017), seems unlikely. The community likely originates from a combination of sources, including groundwater, soil, and atmospheric inputs.

The dynamics of the ghost-rock community are also poorly understood. Ghost-rocks may act as water reservoirs (Champollion et al. 2018) with microbial community composition shifting in response to changing hydrologic conditions, notably during recharge periods. Hydrological boundaries are known to structure the community composition (Smith et al. 2018) and deeper, permanently flooded ghost-rock likely harbor different microbial assemblages less reliant on atmospheric chemosynthesis. Overall, addressing the spatio-temporal variations of ghost-rock microbial communities will need expanded sampling and long-term monitoring.

At geological timescales, microbial activity may have influenced both bedrock weathering, and ghost-rock formation. Ghost-rocks result from the dissolution of soluble bedrock components, and accumulation of insoluble residues, including metal oxides. The presence of metal-interacting pathways in these microbial communities suggest potential interactions between micro-organisms and rock weathering processes.

### Interaction with metals and weathering through karst history

The detection of metal-interacting pathways was partly limited by conservative antiSMASH parameters, and the fragmented nature of the MAGs, which reduce biosynthetic cluster recovery. Similarly, the criteria used to detect potential for EET, requiring a PCC centered on a multiheme cytochrome with at least 10 CX_2_-_4_CH motifs, may underestimate other EET systems. In particular, nanowires (such as OmcS and OmcE in Geobacter; Wang et al. 2019) and flavin-based electron transfer in some Gram positive bacteria (Light et al. 2018) were not investigated. Moreover, the PCC found could be related to non-metal metabolism, such as the DMSO reduction that uses similar system (Gralnick et al. 2006). Thus, cultivation-based approaches to obtain metal reducing/oxidizing isolates could be highly informative in order to complement and validate the putative metal interacting functions that were predicted through bioinformatics. Nevertheless, a significant number of PCC-containing MAGs was recovered, comparable to those from groundwater and sediment enrichments of electroactive micro-organisms (Arbour et al. 2020). Similar cytochrome-based EET systems have been reported in iron-rich caves (Parker et al. 2022), granitic diorite enrichment (Napieralski and Roden 2020) or a marine subseafloor tunnel (Suarez et al. 2023), although methodological differences complicate direct comparison. Overall, these findings suggest that metal-interacting pathways may influence the rates of rock weathering (Wild et al. 2022). Yet, it remains difficult to predict which micro-organisms initially colonize pristine rocks, whether they possess metal-interacting pathways, and how and to which extent these interactions drive rock weathering processes. Incubation experiments in bioreactors using groundwater from a German carbonate karst have shown that *Aquabacterium* spp. may have played a key role in biofilm formation on carbonate rocks, particularly iron-coated rocks (Sharma et al. 2024). In our groundwater dataset, three *Aquabacterium* MAGs were obtained, with the highest quality MAG (SAW104; 92.13% completeness; 3.08% contamination; contig size N50 = 97 849 bp) showing potential for metal oxidation. Such taxa may thus contribute to initial colonization and bioweathering, promoting the formation of ghost-rock, and subsequently being replaced by the distinct heterotrophic ghost-rocks community described here. Indeed, diverse heterotrophic MAGs were observed within ghost-rocks, mainly Acidobacteriota and Pseudomonadota, that may act as metal reducers benefiting from the presence of iron and manganese oxides that constitute the ghost-rocks (Fig. 2; Pisapia et al. 2021). This reduction could solubilize adsorbed elements as substantial trace elements adsorption to iron and manganese oxides is well documented (Tebo et al. 2004, Shi et al. 2021). At the scale of karstic systems, ferromanganese deposits can immobilize trace elements (Frierdich and Catalano 2012), mitigating heavy metals contamination (Newsome et al. 2021). Conversely, dissimilatory reduction of iron oxides can release toxic metal such as adsorbed arsenic from aquifer sediments (Islam et al. 2004). Ghost-rock samples are enriched in redox-sensitive and potentially toxic heavy metals compared to embedded bedrock samples (Fig. 2) and may thus act as dynamic geochemical reservoirs. Microbial metal reduction could occur in anoxic micro-niches, or even under oxic conditions (Ceriotti et al. 2025), releasing adsorbed compounds into groundwater. Given the putative long water residence time of ghost-rocks (Champollion et al. 2018), the presence of metal reducers in ghost-rocks would favor partial dissolution of iron and manganese oxides, and significantly influence trace element mobility within karst aquifer. However, the precise nature of microbial impact on bedrock and ghost-rock weathering remains poorly understood. To elucidate these processes, *in situ* experiments combining geochemical monitoring and metatranscriptomics are needed to directly link microbial activity for microbial activity with mineral transformation.

## Conclusion

The metagenomic analysis of the Sterkfontein karst system reveals highly distinct microbial communities between groundwaters and ghost-rocks, both taxonomically and metabolically. Groundwater communities are dominated by mixotrophic and autotrophic MAGs, primarily belonging to the Gammaproteobacteria, Nitrospirota and Thermoproteota, capable of utilizing ammonium, nitrite or thiosulfate as electron donors, and degrading refractory plant-derived organic matter. By contrast, ghost-rocks host predominantly necromass-degrading heterotrophic MAGs, some of them being putative metal reducers, from the Acidobacteriota and Pseudomonadota phyla, alongside autotrophic Actinobacteriota potentially relying on atmospheric chemosynthesis. The importance of α-glucan metabolism related to glycogen synthesis suggests carbon and energy storage strategies adapted to the oligotrophic and fluctuating hydrological conditions of ghost-rocks. Both ecosystems harbor MAGs with the potential to modify the redox state of metals, suggesting complementary roles in rock colonization and weathering. *Aquabacterium* species in groundwaters may initiate metal oxidation while putative heterotrophic metal reducers in ghost rocks may contribute to the dissolution of the metal oxides. Overall, the study highlights the ecological and geochemical significance of metal-interacting micro-organisms in influencing element cycling, mineral transformation, and the long-term evolution of karstic systems such as Sterkfontein.

## Supplementary Material

fiag047_Supplemental_Files

## Data Availability

All raw sequencing reads and high quality MAGs were deposited on NCBI under the Bioproject PRJNA1298313. The datasets generated and analyzed in the present study are available from the corresponding author.
