# Correction to: Ghost-rocks’ microbiota: metagenomic insights into their influence on the biogeochemistry of karstic cave and groundwater

**DOI:** 10.1093/femsec/fiag088

**Published:** 2026-07-31

## Abstract

In this work, metagenomes from the Artic, Baltic Sea, and Antarctic sea ice were analyzed, revealing insights into genetic diversity, virus-host interactions, and functional roles of viruses in sea ice.

This is a correction to: Guillaume Peugnet, Céline Pisapia, Bénédicte Ménez, Maud Watkinson, Léna Lecourt, Nicolas Peugnet, Julien Bouchez, Laurent Bruxelles, Emmanuelle Gérard, Ghost-rocks’ microbiota: metagenomic insights into their influence on the biogeochemistry of karstic cave and groundwater, *FEMS Microbiology Ecology*, Volume 102, Issue 6, June 2026, fiag047, https://doi.org/10.1093/femsec/fiag047.

The first name of the second co-author has been corrected to read: “Céline Pisapia”.

