# Supplementary material for: Ghost-rocks’ microbiota: metagenomic insights into their influence on the biogeochemistry of karstic cave and groundwater
Source: FEMS Microbiol Ecol. 2026 Jun 10;102(6):fiag047. doi: 10.1093/femsec/fiag047 (PMC13264449; doi:10.1093/femsec/fiag047)

# Supplementary Figures

**Supplementary Figure S1. Abundance of catabolic pathways at the read level** in groundwater samples (W_FC02, W_GC02, W_GC3, W_SL02, W_SL3) and ghost-rock samples (R_FC1, R_FC2, R_SL1), normalized in copies per millions (cpm). Mean abundances for groundwater and ghost-rocks are indicated by horizontal black lines. Pathways are grouped according to the MetaCyc definitions into the following categories: (A) Carboxylate degradation, (B) Aromatic compound degradation, (C) Polysaccharide degradation.


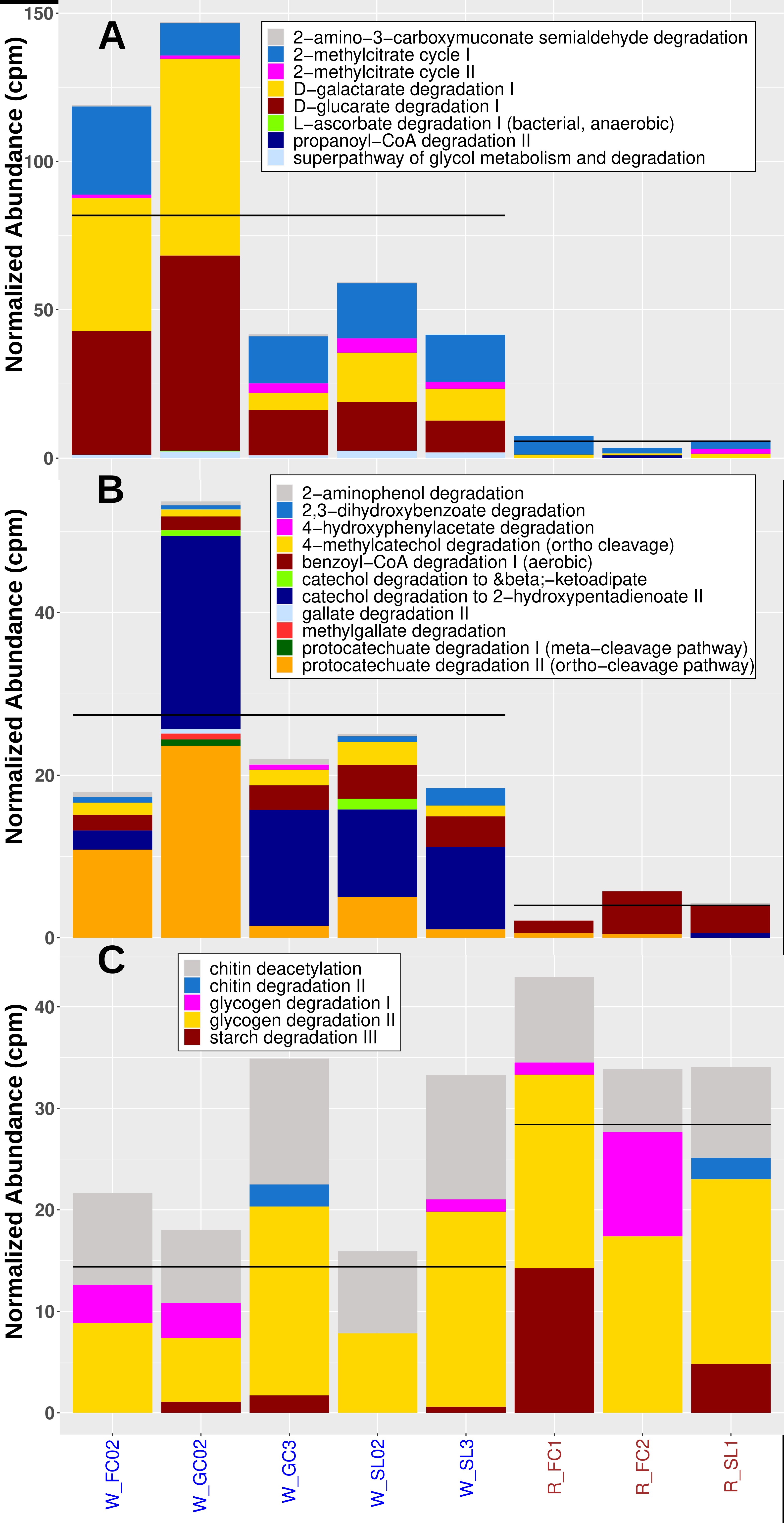


**Supplementary Figure S2.** Comparative analysis of hexuronate degradation operons


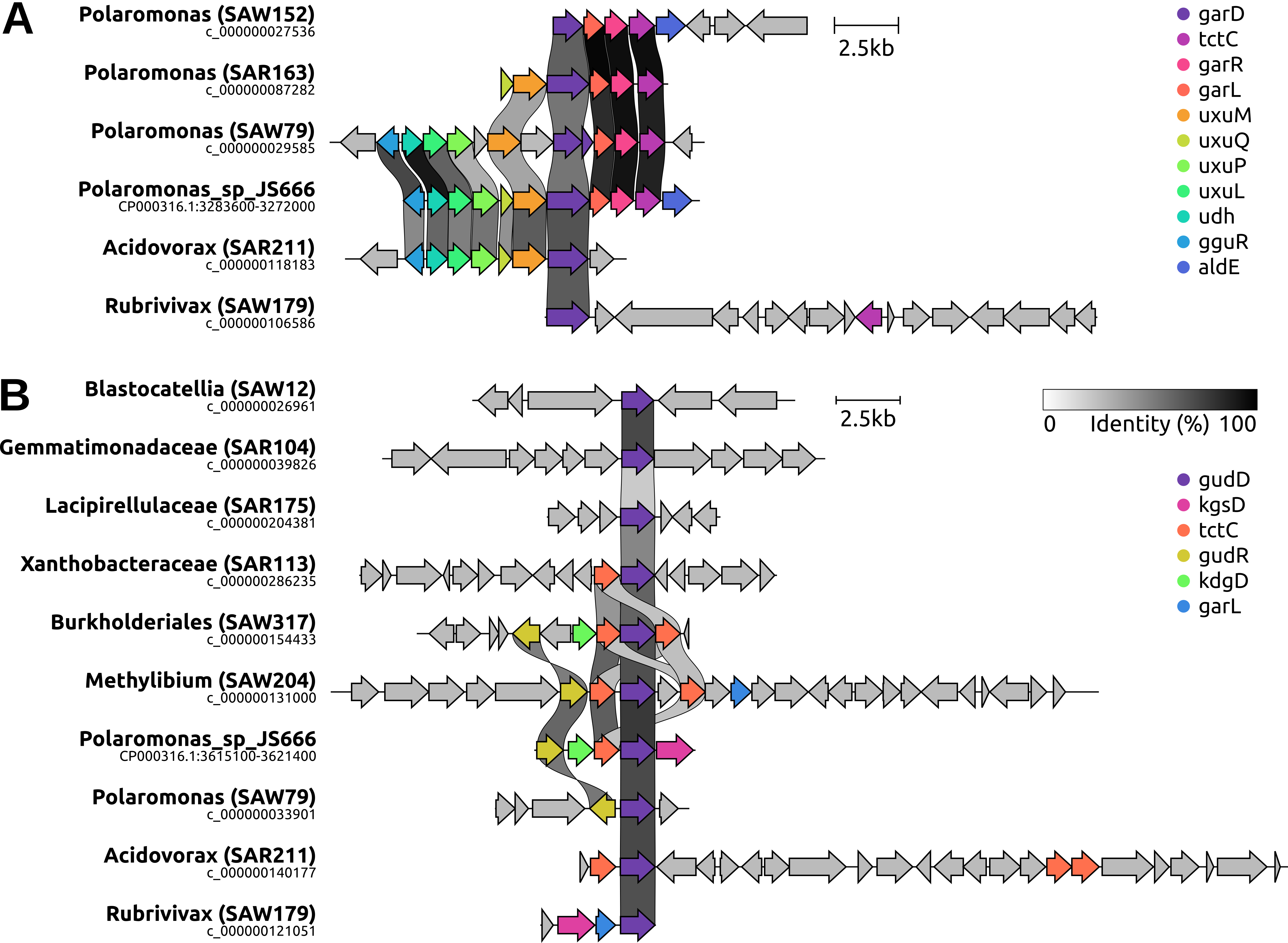


**Supplementary Figure S3.** Number and classification of biosynthetic gene clusters (BGCs) identified in the 273 MAGs


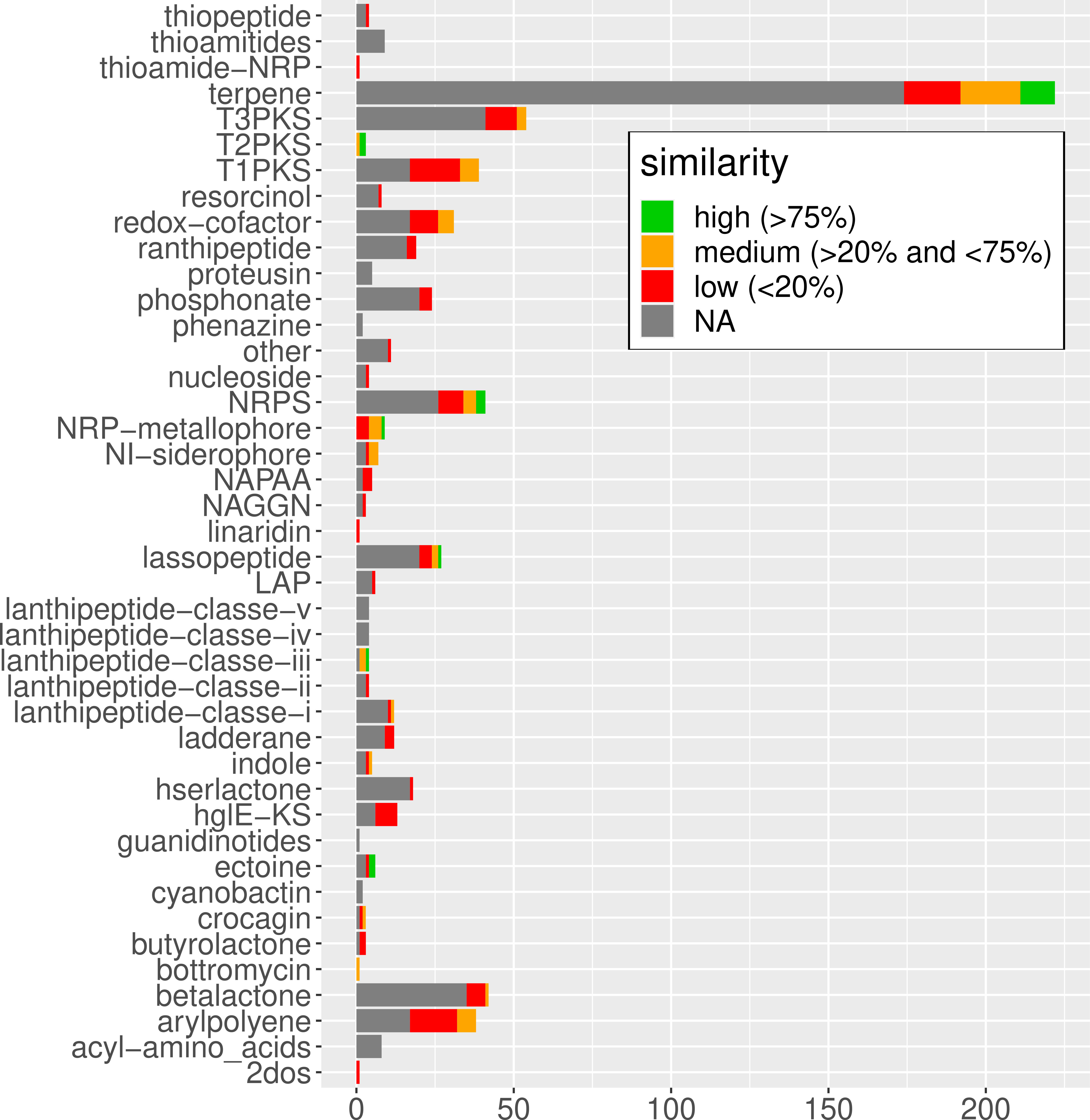


**Supplementary Figure S4.** Mean normalized coverage and number of all biosynthetic gene clusters (BGCs) encoded by each MAG as found by antiSMASH.


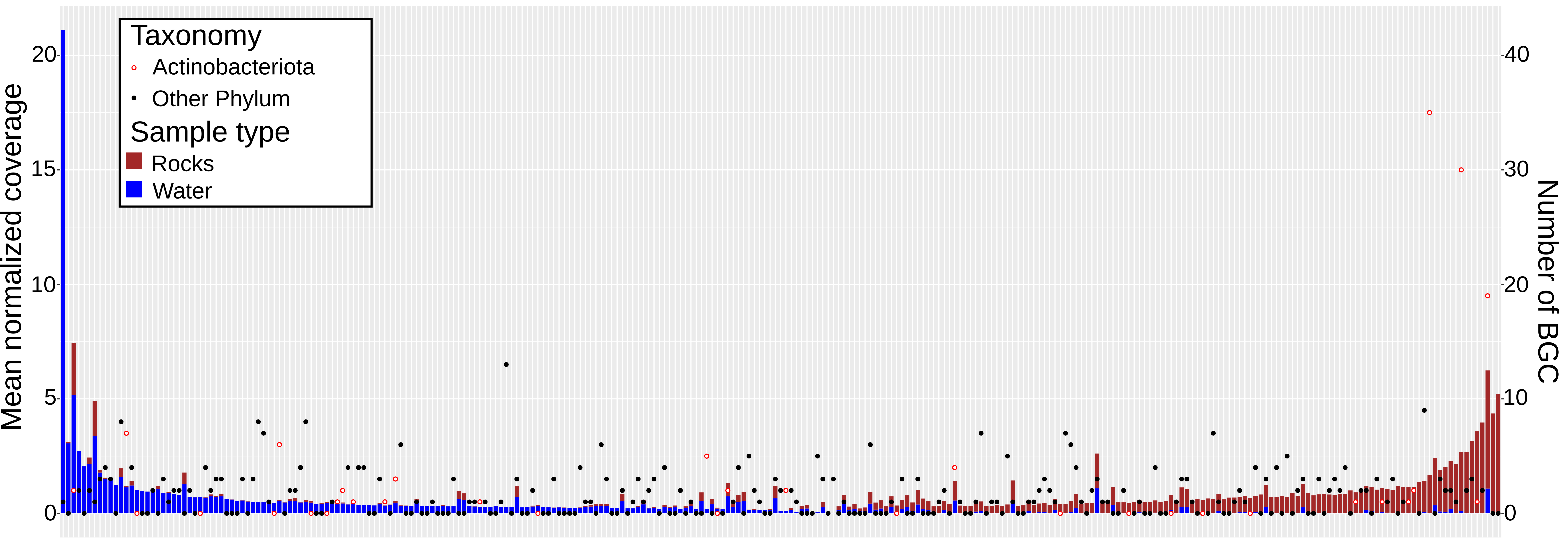

Supplement: fiag047_Supplemental_Files [file fiag047_supplemental_files.zip › Supplementary_images.docx]
